# The shallow domestication bottleneck of strawberry: recovering secondary metabolite-based defenses from wild *Fragaria* species

**DOI:** 10.3389/fpls.2026.1875182

**Published:** 2026-06-24

**Authors:** Sara Postacchini, Patricia Pacheco-Ruiz, Sonia Osorio, José G. Vallarino, Bruno Mezzetti, Luca Mazzoni

**Affiliations:** 1 Department of Agricultural, Food and Environmental Sciences, Università Politecnica delle Marche, Ancona, Italy; 2 Instituto de Hortofruticultura Subtropical y Mediterránea “La Mayora”, Departamento de Biología Molecular y Bioquímica, Universidad de Málaga–Consejo Superior de Investigaciones Científicas (IHSM-UMA-CSIC), Málaga, Spain

**Keywords:** biotic stress, fungicide reduction, genomic resolution, multi-omics, plant defense, secondary metabolites, shallow domestication bottleneck, wild *Fragaria* species

## Abstract

Strawberry (*Fragaria x ananassa*) domestication bottleneck is unique among major fruit crops with a domestication history of merely ~300 years. Unlike wheat, apple, or tomato, whose domestication spans millennia, this shallow bottleneck allows quantification and recovery of the metabolic diversity eroded during selection. The selection for commercial traits, such as color and dimension, inadvertently compromised plant resistance to biotic stresses by reducing key secondary metabolites involved in the defense mechanism. Since the last comprehensive survey of strawberry metabolome, new analytical platforms have substantially expanded the known chemical space of *Fragaria*. This review examines current multi-omics approaches, now aided by the availability of high-quality reference genomes for multiple *Fragaria* species, that enable in-depth characterization of the volatilome by GC-MS, integration of metabolome, transcriptome, and genome data, and high-throughput targeted and untargeted metabolomics. Additionally, this work provides evidence from recent studies on strawberry plants regarding their response to *Botrytis cinerea* and powdery mildew infections through the expression of specific genes, such as *FvPR10.14*, *FaTPS1*, and *FnPR1B*. Furthermore, recent screening of 18 *Fragaria* germplasms identified *F. nilgerrensis* as a resistant species against *B. cinerea*. Notably, genes involved in synthesizing defense compounds, such as terpenes, flavonoids, and ellagitannins, are still found in wild species like *F. chiloensis*, *F. virginiana*, *F. nilgerrensis*, and *F. vesca*. The recent domestication, crossability of wild progenitors, and the current availability of omics tools make *Fragaria* a particularly suitable system for recovering native chemical defenses, providing a sustainable alternative to reliance on fungicides.

## 1 Introduction

Strawberry breeding faces a paradox that few crops share. *Fragaria* × *ananassa* carries one of the heaviest fungicide loads in horticulture, yet its domestication is so recent that the genes lost during selection still circulate in living, crossable wild progenitors. Where wheat domestication unfolded over roughly ten thousand years and apple over four thousand (Cornille et al., 2012), the cultivated strawberry emerged from a single eighteenth-century cross between octoploids *F. chiloensis* and *F. virginiana*, less than three centuries ago. Three hundred years is not enough time to bury a chemical inheritance.

Selection for fruit size, color, and post-harvest performance reshaped the metabolome rather than the genome alone. Concentrations of terpenes, ellagitannins, and specific flavonoids, all linked to constitutive and induced defense, declined in commercial cultivars relative to their wild ancestors, and the magnitude of this loss correlates with susceptibility to biotic stress (Badmi et al., 2023). The economic outcome is visible in a single pathogen: *Botrytis cinerea* remains the principal driver of pre- and post-harvest losses in strawberry production worldwide and the main reason fungicide programmes dominate crop protection (Petrasch et al., 2019).

Eight years ago, Haugeneder et al. (2018) summarized what was then known of the strawberry metabolome and concluded that comprehensive chemical phenotyping was, in practice, beyond reach. The instrumental ceiling has since collapsed. UHPLC coupled to high-resolution mass spectrometry, GC-MS volatilomics with chemometric backbones, mass spectrometry imaging, and haplotype-resolved reference assemblies now operate on routine timelines. The first chromosome-level octoploid assembly of the commercial cultivar ‘Camarosa’ was released in 2019 (Edger et al., 2019); by 2026 the public catalogue holds at least eight haplotype-resolved or telomere-to-telomere assemblies covering both subgenomes and several wild relatives. Multi-omics integration in *Fragaria* has shifted from showcase paper to baseline expectation.

**Figure 1:**
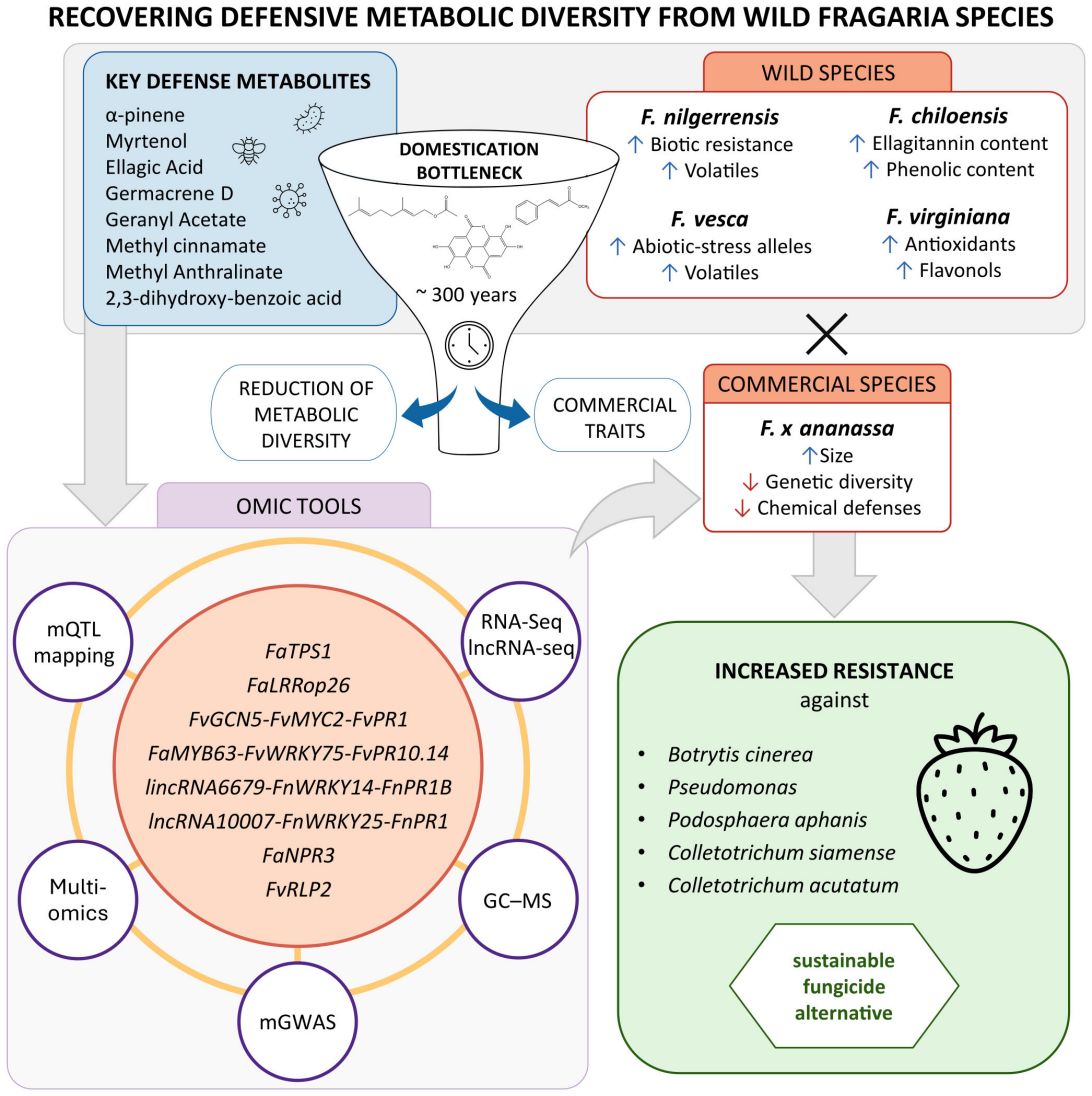
Conceptual overview of the recovery of defensive metabolic diversity in *Fragaria × ananassa* from wild species. Top: wild *Fragaria* species and their characteristic defense-related metabolites. Middle: the ~300-year domestication bottleneck has narrowed metabolic diversity in commercial cultivars through selection for size, colour and shelf life. Bottom: modern omic tools (mQTL mapping, mGWAS, RNA-Seq, lncRNA-Seq, GC-MS, multi-omics) enable the identification of key regulatory genes that can be reintroduced into *F. × ananassa* through crosses with extant wild accessions, reducing fungicide dependence by restoring natural resistance against *Botrytis cinerea*, *Pseudomonas*, *Podosphaera aphanis*, *Colletotrichum siamense* and *C. acutatum*.

This mini-review treats that shift as an opportunity, not a catalogue, and is structured around three propositions. The bottleneck of strawberry domestication is shallow enough to make metabolic loss measurable. The platforms now available are sensitive enough to map that loss to genes, alleles, and tissues. The crossable wild *Fragaria* pool is broad enough to make recovery actionable in real breeding pipelines (**Figure 1**). The remaining question is whether commercial breeding will use these resources before fungicide resistance forces the issue. Relevant literature was identified in PubMed and Web of Science combining *Fragaria* and wild-species names with defense, metabolite, pathogen (*Botrytis*, *Colletotrichum*, powdery mildew) and omics terms (mQTL, mGWAS, multi-omics, reference genome). Priority was given to primary, peer-reviewed studies published after Haugeneder et al. (2018), with foundational earlier work retained where it defines a chemical or genomic baseline; functional validation was favored over correlative association, and all cited records were verified against a resolvable DOI.

## 2 New windows into the strawberry secondary metabolome

Eight years after Haugeneder et al. (2018), four lines of analytical advance have changed what can be asked of the *Fragaria* metabolome.

Genomic resolution is the first, and the most quantitative. The 2019 ‘Camarosa’ assembly catalogued an entire octoploid genome for the first time (Edger et al., 2019); by 2026 the field operates on a stack of haplotype-resolved or telomere-to-telomere references. The list now includes the *F. vesca* v6 ‘Hawaii-4’ assembly with 36,173 annotated genes (Zhou et al., 2023), the gap-free *F.* × *ananassa* assembly of Song et al. (2023), the T2T white-fruited cultivar of Zhang et al. (2024), the chromosome-level *F. nilgerrensis* genome of 270.3 Mb (Zhang et al., 2020), the *F. pentaphylla* draft of Sun et al. (2022), and the recently released ‘Yuexin’ resource of Lu et al. (2025). Jin et al. (2023) crossed the conceptual line by publishing haplotype-resolved assemblies of both octoploid wild progenitors. Mapping reads to subgenome- and haplotype-specific references resolves the noise that limited mQTL detection in the pre-2019 era and turns allele dosage into a tractable variable. Yet the allo-octoploid architecture of *F. × ananassa* (2n = 8x = 56) remains a functional obstacle: four homoeologous subgenomes mean a gene validated in a diploid relative may have redundant or divergent copies in the crop.

Comparative interspecific metabolomics is the second window. Du et al. (2024) profiled 1,008 metabolites across 13 accessions of eight diploid *Fragaria* species, the broadest chemical census of the genus to date. Wang et al. (2025) added a focused *F. nilgerrensis* study, and Kelanne et al. (2025) reconstructed interspecific *F. virginiana* × *F. chiloensis* hybrids to test which traits travel through a deliberate *de novo* cross. Pacheco-Ruiz et al. (2026) extended the same logic to multi-environment European trials. The cumulative effect is a redefinition of the unit of analysis: from cultivar to species, and from species to the wild–cultivated–environment continuum.

Multi-omics with functional causality is the third. Fan et al. (2022) integrated metabolome, transcriptome, and genome data on octoploid accessions to dissect the genetic architecture of strawberry flavor. Porter et al. (2026) closed a long-open mechanistic loop by identifying an enoyl-CoA hydratase as a causal gene for γ-decalactone variation, complementing the *FAD1* work of Oh et al. (2021). Bi et al. (2025) added *FvCOP1* functional knockouts to the validated gene list. What was exceptional before 2018 is now becoming the standard endpoint for a credible discovery paper.

The fourth window is mass spectrometry imaging, and it is almost empty. Enomoto et al. (2018) remains the only robust application of MALDI-IMS to strawberry, with on-tissue derivatization of ABA and OPDA in achene-bearing material. Seven years later no follow-up of comparable scope has appeared. The chemical cartography of the achene–cortex–pith axis remains *terra incognita*, an analytical vacuum surrounded by abundant suitable tissue and explicit physiological hypotheses.

Beneath these four windows, mQTL mapping has matured into a connective infrastructure rather than a stand-alone tool. Pott et al. (2020) reported 309 QTLs for 77 metabolites in cultivated strawberry, with several hotspots concentrated on a small number of linkage groups. Urrutia et al. (2023) extended the approach to volatiles in *F. vesca* and identified the *FanAAMT* family controlling methyl anthranilate. Fan et al. (2022) and Porter et al. (2026) layered transcriptomics and proteomics on the same genetic architecture, moving from statistical association to candidate genes with functional support.

These windows form a connected workflow rather than parallel tracks. mQTL mapping localizes a metabolic trait to a genomic interval (Pott et al., 2020), reference genomes convert that interval into candidate alleles, and multi-omics moves the strongest candidates toward functional validation, as in the flavor-gene framework of Fan et al. (2022) and the *FanAAMT* methyl anthranilate family resolved by Urrutia et al. (2023). Where a discovery instead originates in a single resistant species, as with the *F. nilgerrensis* regulatory modules discussed below, the species genome (Zhang et al., 2020) provides the reference that makes the finding tractable and transferable rather than the route of discovery itself. The Haugeneder ceiling is no longer the operating limit.

## 3 Secondary metabolites as functional defense traits

The literature on strawberry defense has shifted from descriptive correlation to functional validation, and the regulatory layers exposed in the past five years span transcription factors, long non-coding RNAs, cell-wall receptors, and chromatin modifiers. The pattern is clearer when read by pathogen.

Against *Botrytis cinerea*, the core mechanism with the strongest functional support is terpene-mediated antifungal activity. Zhang et al. (2022) showed that *FaTPS1* produces germacrene D, that overexpression in fruit delays *B. cinerea* infection, and that the transcription factor *FaMYC2*, induced by methyl jasmonate, binds the *FaTPS1* promoter. The pathway is gene-to-metabolite-to-phenotype, validated rather than inferred.

A second axis emerged from the resistant background of *F. nilgerrensis*. Guan et al. (2026) screened 18 *Fragaria* germplasm accessions, identified *F. nilgerrensis* as highly resistant, and dissected a non-coding regulatory module in which *lincRNA6679* (a long intergenic non-coding RNA, the intergenic subclass within the broader lncRNA category) upregulates *FnWRKY14*, which in turn activates *FnPR1B*. Activation requires phosphorylation of *FnWRKY14* by the *FnMAPKK4*–*FnMAPK3/6* cascade. The same group, in a companion study, reported the mirror image: *lncRNA10007* is associated with *FnWRKY25*-mediated repression of *FnPR1*, downregulating defense (Guan et al., 2025). The functional symmetry is striking. Two long non-coding RNAs target the same WRKY family in opposite directions, and both pass through the canonical *PR1* output. The regulatory grammar of strawberry immunity is more layered than the protein-coding catalogue suggested.

Chromatin contributes a third layer. Yu et al. (2025) showed that *FvGCN5*, a histone acetyltransferase, enhances *B. cinerea* resistance in *F. vesca* when overexpressed, upregulating *FvMYC2* and *FvPR1*, adding an epigenetic node to the strawberry defense network. Murena et al. (2025) found that elicitor priming (β-aminobutyric acid, β-homoserine, jasmonic acid) across three *F. × ananassa* cultivars produced cultivar-specific metabolic signatures, so priming must be parameterized on commercial backgrounds rather than a generic ‘strawberry’.

Ellagitannins, long described as defense-relevant on correlative grounds, gained mechanistic weight from Grellet-Bournonville et al. (2021). The purified ellagitannin HeT acts as an elicitor of the strawberry response to *Pseudomonas*, an early entry point linking the rich ellagitannin pool of *Fragaria* fruit to receptor-level immune activation.

Against powdery mildew, the dominant axis is the *FaMYB63*–*FvWRKY75*–*FvPR10.14* module described by Jiang et al. (2025). RNAi against *FaMYB63* increases conidiation and lesion size, salicylic acid feeds the pathway, and *FvPR10.14* overexpression suppresses *Podosphaera aphanis* spore germination. Duan et al. (2022) provided complementary transcriptome–metabolome data across three developmental stages and reported the unexpected downregulation of quercetin in mature infected fruit, evidence that flavonoids cannot be invoked as a uniform defense currency without stage-specific qualification.

*Colletotrichum*, historically underrepresented in functional work, finally entered the validated catalogue. Li et al. (2026) showed that *FaLRRop26* responds to *C. siamense* and that CRISPR-Cas9 editing of *FaLRRop26* and its alleles increases lesion size by suppressing lignin monomer biosynthesis (~17% lower lignin at 0 dpi, ~30% versus ~87% accumulation at 4 dpi in mutants versus wild type). Súnico et al. (2024) silenced *FaNPR3* and reduced damage caused by *C. acutatum*, a result consistent with the canonical role of NPR3 as a negative regulator of SA-dependent immunity.

The most striking mechanistic convergence in 2023 is the receptor–cell wall axis. Bai et al. (2023) showed that the *F. vesca* receptor-like protein *FvRLP2* recognizes the *B. cinerea* effectors BcXYG1 and BcPG3 and that overexpression inhibits infection. In the same year, López-Casado et al. (2023) edited the strawberry pectin-modifying enzyme *FaPG1* by CRISPR-Cas9 in cv. ‘Chandler’, primarily to improve fruit firmness, and reported reduced *B. cinerea* damage as a secondary outcome. Plant and pathogen polygalacturonases sit on opposite sides of the same molecular interface; editing the host enzyme weakens the substrate that the pathogen exploits while the host receptor monitors its homologue. The mechanism is honest in both directions.

Lee et al. (2023), via transient RNAi against *FaWRKY29* and *FaWRKY64* in ‘Florida Brilliance’, reduced *Botrytis* disease frequency, enlarging the validated target list along susceptibility-gene lines without adopting the S-gene label.

The cumulative picture is no longer a list of correlations. Strawberry defense is regulated by transcription factors (*MYB63*, *WRKY14*/*25*, *WRKY29*/*64*), long non-coding RNAs operating in opposing directions, cell-wall receptors (*RLP2*), hormonal signaling (JA, SA), and chromatin modifiers (*GCN5*). Three pathogen genera are covered, and a CRISPR-edited proof of concept already exists for a defense-relevant phenotype. Quantifiable, manipulable, and increasingly mechanistic. Two caveats temper this picture. Most of these mechanisms rest on single studies, and functional validation has so far concentrated in diploid models (*F. vesca, F. nilgerrensis*) or in transient octoploid assays rather than stable elite backgrounds. The catalogue is a foundation for hypothesis-driven breeding, not a settled map.

## 4 Wild *Fragaria* species as a recoverable reservoir of defensive metabolic diversity

The defensive chemistry that commercial strawberry lost is, in most cases, still alive in its closest relatives, and the recent literature has begun to put numbers on the gap.

*Fragaria chiloensis*, one of the two octoploid progenitors, is the clearest example. Chamorro et al. (2025) compared Chilean wild ecotypes against the cultivars ‘Albion’ and ‘San Andreas’ and found roughly 37% higher antioxidant capacity by DPPH and 13% higher total phenolic content in the wild material. The class-specific differences are sharper. Ellagic acid concentration was 2.5 times higher and cyanidin-3-glucoside 2.6 times higher in *F. chiloensis* subsp. *chiloensis* than in the commercial cultivars. Two centuries of selection for size and shelf life translated into a measurable contraction of two of the most pharmacologically and ecologically relevant compound classes in the species.

*Fragaria virginiana*, the second octoploid progenitor, carries the same direction of effect. Wang and Lewers (2007) reported substantially higher total antioxidant capacity, total phenolics, and total anthocyanins in *F. virginiana* accessions compared with both *F.* × *ananassa* and *F. chiloensis* ecotypes, with flavonol content also exceeding the cultivated reference. The original work predates the modern genomic toolset but defines the chemical space that subsequent multi-omics analyses now interrogate at allele resolution. Similarly, Diamanti et al. (2014) reported improvements in fruit sensorial and nutritional quality from a strawberry interspecific backcross program employing this progenitor.

*Fragaria vesca* contributes a different kind of value. The diploid woodland strawberry produces methyl anthranilate, a volatile largely absent from commercial fruit and responsible for the distinctive woody-grape aroma, through the *FanAAMT* family characterized by Urrutia et al. (2023). Methyl anthranilate is not a defense compound by historical convention, but its antifungal activity *in vitro* positions it at the intersection of flavor and protection (Chambers et al., 2013), an unusual combination in breeding terms because both endpoints align in the same direction. Kanbar et al. (2024) added an abiotic stress dimension by linking the Cold Box Factor 4 transcription factor and the dehydrin Xero2 to cold acclimation in specific *F. vesca* accessions, raising the prospect of introgressing wild alleles to address freezing damage in highly sensitive elite backgrounds.

*Fragaria nilgerrensis*, the diploid white-fruited species, has shifted from genomic curiosity to functional reservoir. Duan et al. (2025) combined transcriptomics with volatilome profiling and identified MYB transcription factors positively correlated with stress-related metabolites, including 2,3-dihydroxy-benzoic acid and geranyl acetate, both present in trace amounts or downregulated in cultivated strawberry. Specific candidate genes (FxaYL_542g0723070, FxaYL_512g0659290) were associated with phenylpropanoid and terpenoid pathways through expression-metabolite correlations that now require functional validation. The 270.3 Mb chromosome-level genome of *F. nilgerrensis* (Zhang et al., 2020) anchors these findings to a tractable reference and connects them to the comparative diploid chemistry of Du et al. (2024).

*De novo* species reconstruction is the most direct test of the recovery thesis. Kelanne et al. (2025) crossed elite germplasms of *F. virginiana* subsp. *virginiana* and *F. chiloensis* and measured the volatile profile of the resulting hybrid progenies. Total aldehydes increased by 24%, esters, acetates, and volatile acids approximately doubled, and γ-aminobutyric acid (GABA), a stress-associated metabolite linked to post-harvest quality, accumulated above commercial reference levels. The experimental crossability of the wild progenitors is not theoretical. The hybrids exist, the chemistry shifts in the predicted direction, and the agronomic phenotype is acceptable.

Across the four species the direction is consistent: wild relatives retain phenolic, antioxidant, volatile, and resistance traits eroded in the cultivar. The shallow domestication bottleneck (**Figure 1**) is not a metaphor here; it is the operational reason wild–cultivated crosses still produce viable, fertile progenies and the recovery agenda is breeding-actionable rather than confined to comparative biochemistry.

## 5 Concluding remarks and future perspectives

The arc of this review is short and uncomfortable. The bottleneck is shallow, the wild reservoir is alive, the analytical platforms are mature, and the mechanistic catalogue is no longer empty. What is missing is targeted use of these resources in the breeding programs that supply the global strawberry market.

Four gaps deserve explicit declaration. The biosynthetic genes for ellagitannins and proanthocyanidins, the chemical classes most often invoked as defense-relevant in *Fragaria*, lack functional validation in a defense context for the period 2024–2026; no overexpression, RNAi, or CRISPR study has yet closed that loop. Stable transformation of elite octoploid backgrounds remains recalcitrant and limits validation in commercial germplasm. Allelic complexity persists; even with telomere-to-telomere assemblies, resolving the contribution of each homoeologous copy to a quantitative trait is a bioinformatic challenge rather than a solved problem. Microbiome–metabolome integration in the context of strawberry defense is an early-stage field, with few published datasets and no consensus pipeline.

Genome editing in *Fragaria* has crossed the proof-of-concept threshold but not the production one. Lee et al. (2023) validated *FaWRKY29* and *FaWRKY64* as facilitators of *B. cinerea* infection through transient RNAi; López-Casado et al. (2023) reduced *B. cinerea* damage as a secondary outcome of *FaPG1* CRISPR editing; Rai et al. (2025) edited *FvMYB46* and altered flavanol and lignin profiles in anthers; Li et al. (2026) edited *FaLRRop26* in an octoploid background and demonstrated lignin-mediated resistance to *C. siamense*. Sun et al. (2025) reported potential reversion of CRISPR edits in octoploid ‘Honeoye’, with the mutant allele frequency at the target locus dropping from 70.2% at three months to 43.7% at six months, an issue the field has yet to resolve. The honest summary is that the technology works in *Fragaria*, that the scientific path to defense-relevant edits is open, and that genetic stability in the octoploid context is still under negotiation.

Iqbal et al. (2025) framed the strategic question for breeding within a Breeding for Integrated Pest Management (B-IPM) framework, screening *F. vesca* genotypes simultaneously for resistance to *B. cinerea* and *C. acutatum* and for facilitation of the biocontrol agent *Aureobasidium pullulans*. Pathogen resistance and biocontrol facilitation segregated independently in their material, indicating that both can be optimized in parallel rather than as a trade-off. Metabolomic-assisted breeding for phytosanitary endpoints, mGWAS designs targeted at resistance-marker metabolites such as agrimoniin and germacrene D, and the integration of mass spectrometry into speed-breeding cycles are the most tractable next moves, building on the breeding-value framework that places fruit metabolome variation at the center of strawberry improvement (Senger et al., 2022).

The reservoir exists, the tools exist, the regulatory genes are being validated, and the crossable wild species are sitting in living collections. The remaining variable is whether the breeding sector will use them before fungicide resistance closes the window. Three centuries of domestication can be reopened in a decade. The decision is not technical.
